# Adalimumab biosimilar ABP 501 is equally effective and safe in long-term management of inflammatory bowel diseases patients when used as first biologic treatment or as replace of the ADA originator for a non-medical reason

**DOI:** 10.3389/fgstr.2023.1218228

**Published:** 2023-10-23

**Authors:** Giammarco Mocci, Arianna Cingolani, Giorgia Orrù, Carla Felice, Francesca Maria Onidi, Gianmarco Lombardi, Davide Checchin, Raffaele Colucci, Laurino Grossi, Antonio Ferronato, Chiara Rocchi, Marta Ascolani, Paolo Usai Satta, Lucia Fanini, Stefano Pilati, Antonio Tursi

**Affiliations:** ^1^ Division of Gastroenterology, ARNAS “G. Brotzu”, Cagliari, Italy; ^2^ Division of Gastroenterology and Digestive Endoscopy, “Carlo Poma” Hospital, ASST, Mantova, Mantua, Italy; ^3^ Presidio Policlinico Universitario of Monserrato, Cagliari, Italy; ^4^ Division of Internal Medicine, “Ca’ Foncello” University Hospital, Treviso, Italy; ^5^ Division of Nephrology, Verona University Hospital, Verona, Italy; ^6^ Division of Gastroenterology, Dell’Angelo Hospital, Venice, Italy; ^7^ Digestive Endoscopy Unit, “S. Matteo degli Infermi” Hospital, Spoleto, Italy; ^8^ Division of Gastroenterology, “S Spirito” Hospital, “G. d’Annunzio” University, Pescara, Italy; ^9^ Digestive Endoscopy Unit, Santorso Hospital, ULSS “Pedemontana”, Santorso, Italy; ^10^ Division of Gastroenterology and Digestive Endoscopy, “Mater Olbia” Hospital, Olbia, Italy; ^11^ Division of Gastroenterology, ”Ca’ Foncello” University Hospital, Treviso, Italy; ^12^ Division of Gastroenterology, Foligno Hospital, Foligno, Italy; ^13^ Territorial Gastroenterolgy Service, ASL BAT, Andria, Italy; ^14^ School of Medicine, Department of Medical and Surgical Sciences, Catholic University, Rome, Italy

**Keywords:** adalimumab, ABP 501, biosimilar, Crohn’s disease, ulcerative colitis

## Abstract

**Objective:**

Biosimilars represent a new opportunity for inflammatory bowel disease (IBD) treatment and economic sustainability of therapies. This study aimed to evaluate the efficacy and long-term safety of the adalimumab biosimilar ABP 501 in biologic-naïve vs. biologic-switched IBD patients.

**Methods:**

A retrospective observational study was conducted using a database of patients with IBD treated with ABP 501, biologic-naïve or switched from the original, at eight IBD centers. We included adult patients with at least one year of follow-up. The primary objective of this study was to assess the efficacy (persistence) and safety (adverse event rate) of ABP 501 therapy.

**Results:**

A total of 118 patients with IBD were included in the analysis: 84 patients with Crohn’s disease (CD) (39 women, 45 men, mean age 40.4 ± 14.3 years; 33% biologic-naïve) and 34 patients with ulcerative Colitis (UC) (16 women, 18 men, mean age 38.9 ± 14.9 years; 61.8% biologic-naïve). Regarding the primary endpoint, no difference was observed in the efficacy between biologic-naïve patients and patients with Adalimumab (ADA) originator replacement for non-medical reasons in terms of long-term persistence. However, ABP 501 showed a higher percentage of sustained clinical remission at 2 years in patients with CD (64 patients, 77%) than in those with UC (15 patients, 45.5%; p=0.00091). Nine patients (six with CD and three with UC) experienced adverse events that led to drug discontinuation in three.

**Conclusions:**

APB 501 showed a good safety and efficacy profile in maintaining clinical response at 2 years in patients with IBD, both as a treatment-naïve and as a replacement for ADA originator for non-medical reasons.

## Introduction

Biosimilars have presented a new possibility in treating inflammatory bowel disease (IBD) since their entry into the market several years ago after the originators’ patent expired (1).

In 2013, CT-P13 was the first infliximab biosimilar approved with all the therapeutic indications of the reference product, thereafter, several biosimilars entered common clinical practice for IBD (2).

The European Unit patents on the adalimumab (ADA) originator (Humira^®^, Abbvie, USA) expired in 2018, and ADA biosimilars with the same indications as the originator are currently available (ABP 501, Amgevita^®^, and Solymbic^®^, Amgen, USA; SB5, and Imraldi^®^, Bio-Denmark, Denmark Samsung Bioepis, South Korea; FKB327 and Hulio^®^, Mylan, USA; Fujifilm Kyowa Kyrin Biologics, Japan; GP2017 and Hyrimoz^®^, Sandoz, Germany; and BI 695501, Cyltezo^®^, Germany) (3).

ADA biosimilars currently approved by the European Medicine Agency (EMA) show negligible and preclinical data would indicate the same biological effects and similar pharmacological characteristics as the originator (4). Moreover, preclinical studies and clinical trials in other immune-mediated diseases, such as rheumatoid arthritis or plaque psoriasis, support switching from the adalimumab originator to a biosimilar in patients with IBD. Based on the current regulatory guidance form the EMA and the evidence about efficacy and safety of biosimilars in IBD patients, the European Crohn’s and Colitis Organization (ECCO) made relevant statements to summarise their shared position (5). In particular, in the absence of data from real-life experiences or large clinical trials, switching a patient with IBD from an ADA originator to an ADA biosimilar should only be performed after clinical evaluation (3). Moreover, ‘automatic substitution’ (a practice that allows a pharmacist or other healthcare professional to replace a branded drug prescribed by a doctor with a generic drug without consulting a prescribing specialist) of biological drugs is discouraged by IBD expert associations (5). It must always be authorized by a clinician.

ADA ABP 501 is approved by the Food and Drug Administration (FDA) and EMA (6) for the same indications as that of Humira^®^. In Europe, ABP 501 has been approved for moderate-to-severe hidradenitis suppurativa and adult non-infectious intermediate, posterior, and panuveitis (6).

Looking at the existing literature, most of data regarding safety and efficacy of ABP 501 are currently derived almost exclusively from large randomized clinical trials of non-gastrointestinal immune-mediated diseases (4). However, data comparing ABP 501 and its ADA originator (HumiraTM) in IBD patients are still lacking (7). In particular, comparative analyses of the efficacy of maintaining remission after the replacement of the originator for non-medical reasons are limited (8).

The aim of this study was to assess the role of ADA ABP 501 from a different perspective, namely, whether there is a difference when using this ADA biosimilar as the first choice or as a switch from an ADA originator for non-medical reasons.

## Materials and methods

A multicenter retrospective observational study was performed in nine Italian nontertiary IBD centers, and patients, both naïve and switched from originator adalimumab, who underwent ADA ABP 501 therapy, between January 1, 2018, and December 31, 2020, were selected.

Eligible patients included outpatient men and women, ≥18 years, diagnosed with ulcerative colitis (UC) or Crohn’s disease (CD), and with at least 6 months of follow-up. Data collected included demographic features (age and sex), smoking habits, IBD-related clinical characteristics (disease duration, comorbidities, and previous immunosuppression therapy), extent of the disease (according to the Montreal classification) (9), disease activity (defined as Mayo clinical partial >2 for patients with UC (10), or Harvey Bradshaw Index (HBI) >5 for patients with CD) (11), C-reactive protein (CRP), fecal calprotectin (FC) at baseline and during follow-up, and adverse events during follow-up. All patient data were anonymized and collected from a common database for analysis.

Patients were included in the study after testing negative for *Mycobacterium tuberculosis* infection and active hepatitis B. The ADA biosimilar ABP 501 was administered subcutaneously at a dose of 40 mg every two weeks both in biologic-naïve patients and in patients replacing the ADA originator. None of the patients who were treated with a weekly dose of ADA originator were enrolled. The need to switch to other biological agents or the addition of concomitant medications for the treatment of IBD was considered a failure.

Written informed consent was obtained from all patients at the time of ADA ABP 501 prescription, both naïve and non-medical-reason-switched. This study was conducted in accordance with the clinical practice guidelines of the Declaration of Helsinki. Ethics committee approval was obtained by “Brotzu” Hospital (Cagliari, Italy, PROT. PG/2021/10115).

The primary objective of this study was to evaluate whether there was a difference in long-term treatment persistence (up until the 24^th^ month of treatment) in patients with IBD treated with ADA ABP 501 as the first biologic treatment versus as a replacement for the ADA originator for non-medical reasons as a cost reduction measure.

Secondary objectives were used to assess any difference in terms of: sustained clinical response to ABP 501 therapy (defined as ongoing ABP 501 treatment at the end of the follow-up period), sustained a clinical response to ABP 501 therapy (defined as ongoing ABP 501 treatment at the end of follow-up in CD versus UC patients), rate of steroid-free clinical remission (changes in FC and PCR during the follow-up compared to baseline), and safety profile of the drug in terms of the rate of adverse events occurring during therapy with biosimilar ABP 501.

### Statistical analysis

Quantitative variables are reported as mean and standard deviation (SD) if normally distributed, or median and interquartile range (IQR) if skewed. Categorical variables are reported as frequencies and percentages. Quantitative variables were compared using Student’s t-test or the Mann-Whitney U test, as appropriate and categorical variables were compared using the chi-squared test.

Kaplan-Meier survival analysis was used to estimate the survival rate of sustained clinical responses in the entire study population. Stratified analyses according to IBD type, naïve to anti-TNF therapy, and switching from an originator anti-TNF to a biosimilar were then conducted. The log-rank test was used to compare survival curves.

For sensitivity analysis, we used a Cox regression model to derive unadjusted and multivariable-adjusted hazard ratios (HRs) and 95% confidence intervals (95% CIs) for the association between the exposures of interest and the main outcome. The following covariates were assessed in the multivariate model: age, sex, smoking habits, comorbidities, and IBD age.

For analysis and data calculation, we used the R software (version 3.4.4, R Foundation for Statistical Computing Platform). Statistical significance was defined as a two-tailed p value <0.05.

## Results

A total of 118 IBD patients were included in the analysis: 84 CD patients (39 F, 45 M, mean age 40.4 ± 14.3 years; 33% naïve to biologics) and 34 UC patients (16 F, 18 M, mean age 38.9 ± 14.9 years; 61.8% naïve to biologics). Overall, 49 patients (40.7%) were naïve to biologics.

**Table 1** shows baseline demographic and clinical data.

**Table 1:** Patients features at baseline.

	OVERALL n=118	UC n=34	CD n=84	P value
**Gender, male n (%)**	63 (53.4)	18 (52.9)	45 (53.6)	1.000
**Age, yr, mean**	40.0 (14.4)	38.9 (14.9)	40.4 (14.3)	0.611
**Disease duration, yr, median (IQR)**	6.8 (2.5, 11.4)	7.0 (2.3, 11.3)	6.0 (2.7, 11.4)	0.652
**Comorbidities**	32 (30.2)	13 (38.2)	19 (26.4)	0.311
**Appendectomy, n(%)**	16 (13.6)	3 (8.8)	13 (15.5)	0.339
Concomitant therapy				
Mesalazine	90 (76.9)	31 (91.2)	59 (71.1)	0.036
Steroid	104 (88.1)	32 (94.1)	72 (85.7)	0.335
Thiopurine	46 (39.0)	13 (38.2)	33 (39.3)	1.000
Methotrexate	5 (4.2)	1 (2.9)	4 (4.8)	1.000
**Naïve to biologics, n (%)**	48 (40.7)	21 (61.8)	28 (33.3)	0.500
**Switched, n(%)**	70 (59.3)	13 (38.2)	55 (66.6)	0.002
Montreal classification UC				
Left sided		11 (32.3)		
Extensive		23 (67.7)		
Montreal classification CD				
Isolated ileal			19 (22.6)	
Isolated colonic			9 (10.7)	
Ileocolonic			50 (59.5)	
Isolated UGI			4 (4.8)	
Perianal disease			2 (2.4)	
Behavior				
Non stricturing – non penetrating			38 (45.2)	
Stricturing			30 (35.8)	
Penetrating			16 (19.0)	
**Median partial Mayo score**		4 (5)		
**Median endoscopic Mayo subscore**		1.7 (2)		
**Median HBI**			5 (18)	
**Median SES CD**			10 (12)	
**CRP, median (IQR)**		0.3 (0.9-0.1)	0.3 (0.7-0.1)	0.706
**Calprotectin, median, μg/g (IQR)**		319 (661-218)	159.0 (250-183)	0.221

**Figure 1:**
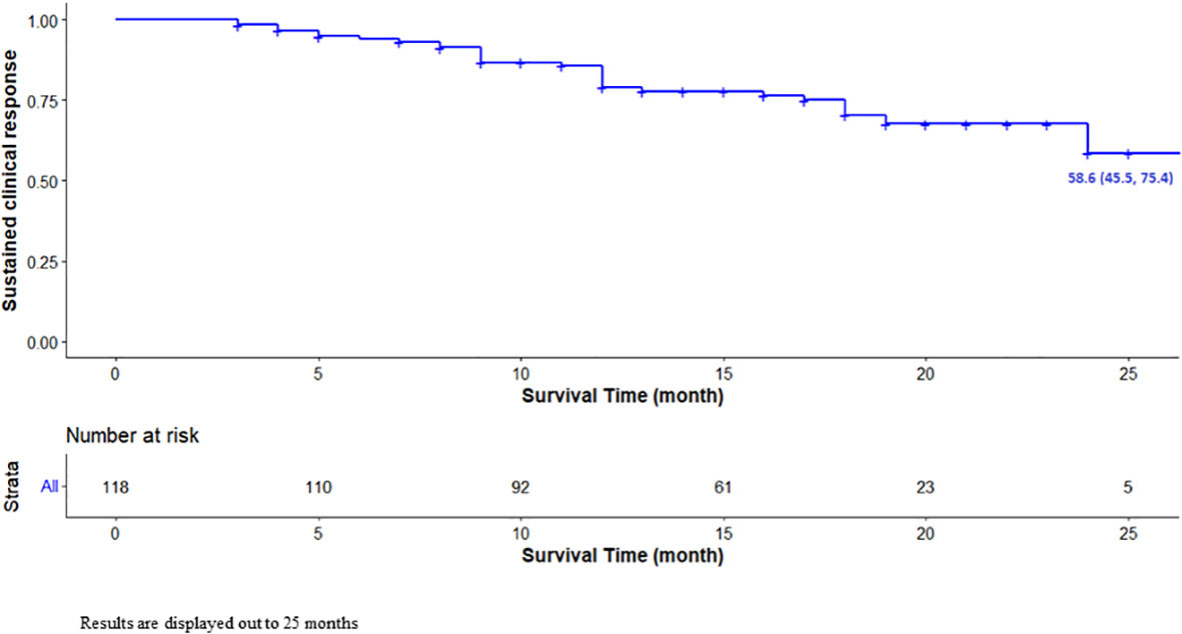
Unadjusted Kaplan-Meier survival curve for sustained clinical response. Results are displayed out to 25 months.

**Figure 2:**
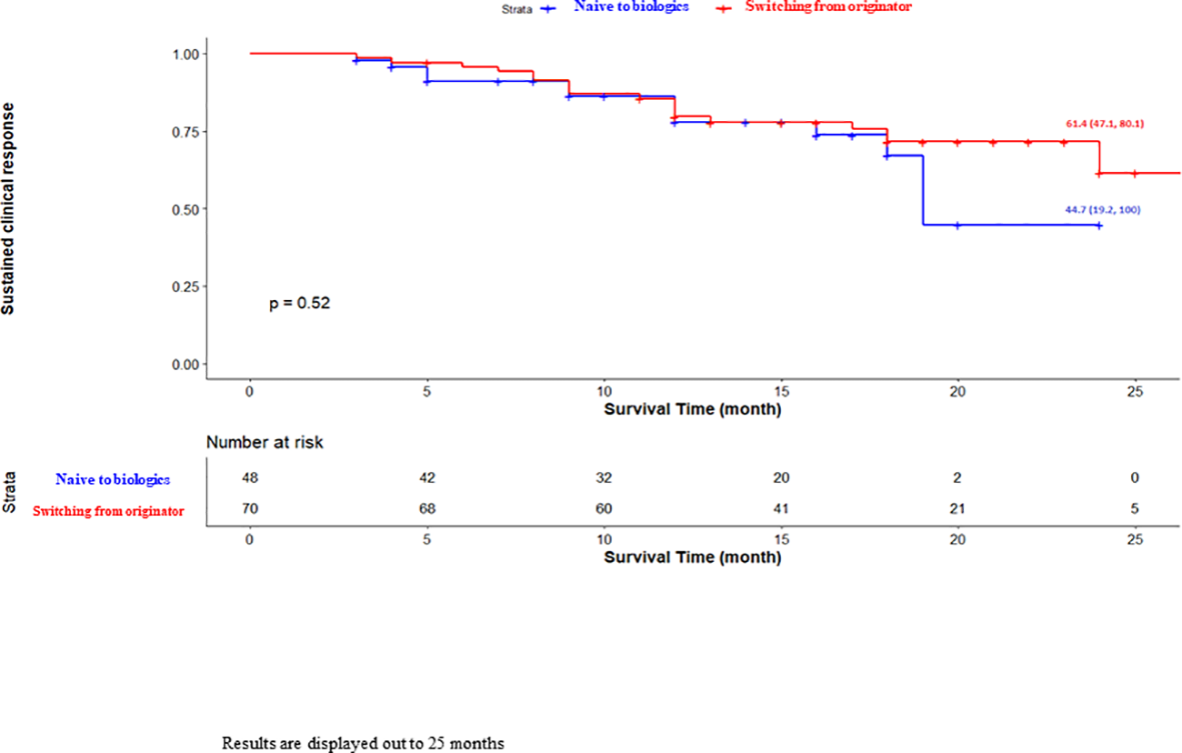
Unadjusted Kaplan-Meier survival curve for sustained clinical response based on naïve to biologics. Results are displayed out to 25 months.

Overall, ABP 501 showed a high percentage of sustained clinical remission at two years (69/118 patients, 58.6%; **Figure 1**). Regarding the primary endpoint, no difference was observed in the efficacy between biologic-naïve patients and patients with ADA originator replacement for non-medical reasons in terms of long-term persistence (**Figure 2**).

**Figure 3:**
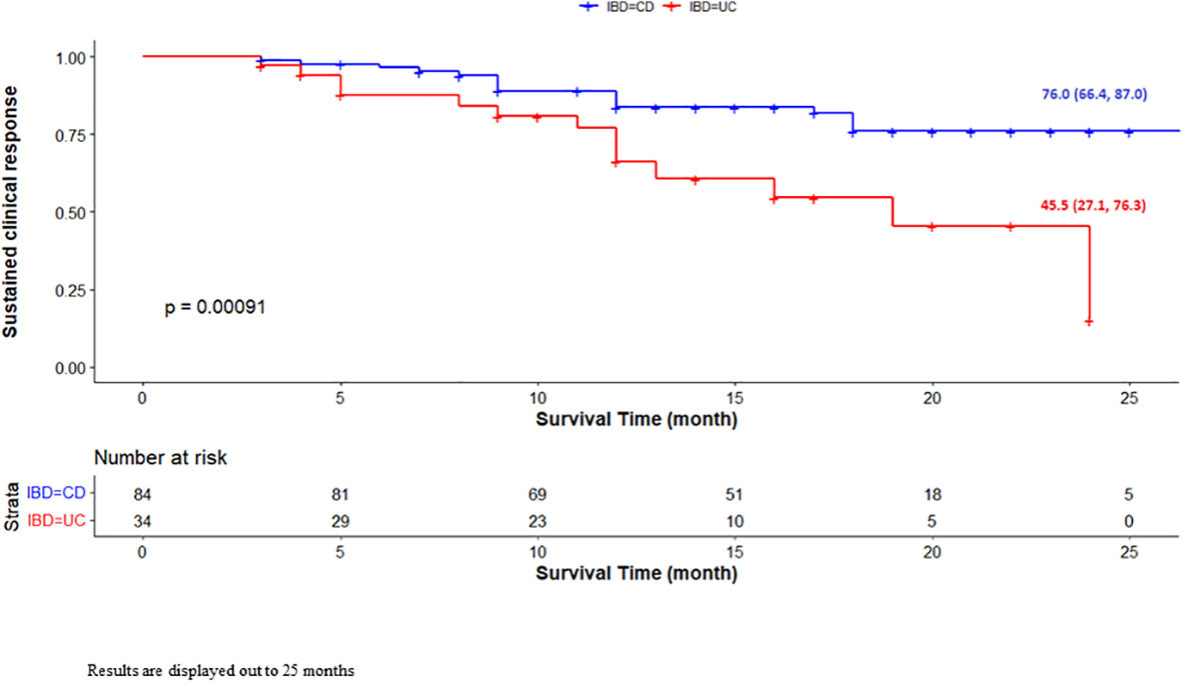
Unadjusted Kaplan-Meier survival curve for sustained clinical response based on IBD type. Results are displayed out to 25 months.

Regarding the secondary endpoints, ABP 501 showed a higher percentage of sustained clinical remission at 2 years in patients with CD (64/84 patients, 76%) than in those with UC (15/34 patients, 45.5%; p=0.00091, **Figure 3**). No factors appeared to influence the sustained clinical response to ABP 501 therapy, including concomitant use of immunosuppressive drugs and extraintestinal manifestations.

Three patients with UC needed ABP 501 optimization (2.5%).

Steroid-free clinical remission at 2 years was achieved in 81/118 patients (68.6%), with a significant difference between UC and CD (20/34 patients, 58.8% vs. 61/84 patients, 72.6%, respectively; p=0.0008).

FC dropped significantly at 24 months compared to baseline. In particular, it dropped from median 319 μg to 128 μg in UC and from 159 μg to 87 in CD (p=0.001 and p=0.02, respectively).

Finally, nine patients (7.62%, six with CD and three with UC), experienced adverse events during follow-up. Three patients (2.54%) discontinued treatment: one UC patient with paradoxical worsening of rectal bleeding, one CD patient with tonsillitis, and one CD patient with paradoxical psoriasis at the injection site.

## Discussion

Although biosimilars are drugs that are similar but not 100% identical to the originator, they may give the opportunity to use drugs that are less expensive with the same efficacy (12). The majority of ADA biosimilars currently available in the market seem to be equivalent in terms of efficacy and safety (13). From a clinical point of view, the most interesting results come from studies analyzing the equivalence of ADA biosimilars to the ADA originator. For example, the ADA GP2017 and ADA originators have been found to be equivalent in terms of efficacy and safety in patients with IBD (14). Clinical data on the equivalence of ABP 501 to the ADA originator are currently derived almost exclusively from large randomized clinical trials of non-gastrointestinal immune-mediated diseases (4) and a previous study found ABP 501 to be effective and safe in Crohn’s disease (7). In contrast, comparative analyses of the efficacy of maintaining remission after the replacement of the originator for non-medical reasons are limited (8).

The present study investigated the role of ADA ABP 501 from a different perspective, namely, whether there is a difference when using this ADA biosimilar as the first choice or as a switch from an ADA originator for non-medical reasons. We did not find any difference when using ADA ABP 501 according to these indications during the long-term (2-years) follow-up. Moreover, this drug was safe because adverse events AEs (in particular, AEs leading to treatment discontinuation) were very low, which is in line with other recent reports (11–15). The most frequent AE observed was hitch/pain at the injection site. A possible explanation could be that this biosimilar contain sodium citrate, a compound that may be responsible for somewhat more pain (16). However, taken together, these results led to the conclusion that ADA ABP 501 may be safely prescribed for every indication without any significant difference in terms of clinical response.

However, a significant difference was found between patients with UC and CD treated with ADA ABP501. Although safety was similar in the two diseases, long-term remission with ADA ABP501 was higher in CD patients than in UC patients. Therefore, this study confirms that ADA, both originator and biosimilar, works better in CD than in UC (11–19), and that this must be kept in mind by clinicians when choosing the right biologic for the treatment of UC (20).

Another important finding is that these patients not infrequently require optimization of the dosage to maintain remission. The optimization rate was about double that required in naϊve patients treated with ADA biosimilars (13) and similar to that occurring in other experiences (7, 18, 19). All these data seem to show that patients who replaced the originator with ADA biosimilars for a non-medical reason often need dose escalation, which may impact the therapy’s burden and make the biosimilars less attractive.

Finally, despite the switch from the originator to the biosimilar has been made for non-medical reasons, overall the patients accepted this change. In this regard, as also suggested in the ECCO recommendations (5), communications by IBD nurse and physicians on the equivalence of biosimilars compared to the originator was crucial.

The strength of this study lies in the long-term follow-up (24 months compared with 6-12 months for the current studies available) and its multicenter nature, which allowed us to obtain real-life data from several centers across Italy using ADA ABP 501, limiting single-center bias and obtaining a representative population.

The limitations of the study are its small sample size and retrospective nature, which poses the risk of bias regarding the “selection” of patients and the interpretation of side effects and did not allow us to have the same timing in the endoscopic follow-up, resulting in insufficient endoscopic data.

In conclusion, ABP 501 showed excellent two-year persistence and maintenance of clinical response, and a low rate of serious or discontinuing adverse effects in patients naïve to biologics or in patients switching from the ADA originator for non-medical reasons. These results suggest that switching to ABP 501 is a safe choice in this particular scenario. This study also confirmed that ADA ABP 501, as well as ADA originator and other ADA biosimilars, works better in patients with CD than in those with UC, suggesting that clinicians should carefully evaluate whether to use ADA (both originator and biosimilar) in UC.

## Data availability statement

The raw data supporting the conclusions of this article will be made available by the authors, without undue reservation.

## Ethics statement

The studies involving humans were approved by Comitato etico Azienda Ospedaliera Universitaria di Cagliari; PROT.PG/2021/10115. The studies were conducted in accordance with the local legislation and institutional requirements. Written informed consent for participation was not required from the participants or the participants’ legal guardians/next of kin in accordance with the national legislation and institutional requirements.

## Author contributions

GM and AT planned and conducted the study; GM, AC, GO, CF, FO, GL, DC, RC, LG, AF, CR, MA, PS, LF, SP, and AT collected the data; GM, AT and CF drafted the manuscript; GM, AC, GO, CF, FO, GL, DC, RC, LG, AF, CR, MA, PS, LF, SP, and AT approved the final draft submitted. All authors have read and agreed to the published version of the manuscript.
